# A weighted relative difference accumulation algorithm for dynamic metabolomics data: long-term elevated bile acids are risk factors for hepatocellular carcinoma

**DOI:** 10.1038/srep08984

**Published:** 2015-03-11

**Authors:** Weijian Zhang, Lina Zhou, Peiyuan Yin, Jinbing Wang, Xin Lu, Xiaomei Wang, Jianguo Chen, Xiaohui Lin, Guowang Xu

**Affiliations:** 1 School of Computer Science & Technology, Dalian University of Technology, Dalian, China; 2 Key Laboratory of Separation Science for Analytical Chemistry, Dalian Institute of Chemical Physics, Chinese Academy of Sciences, Dalian, China; 3 Qidong Liver Cancer Institute, Qidong 226200, China

## Abstract

Dynamic metabolomics studies can provide a systematic view of the metabolic trajectory during disease development and drug treatment and reveal the nature of biological processes at metabolic level. To extract important information in a systematic time dimension rather than at isolated time points, a weighted method based on the means and variations along the time points was proposed and first applied to previously published rat model data. The method was subsequently extended and applied to prospective metabolomics data analysis of hepatocellular carcinoma (HCC). Permutation was employed for noise filtering and false discovery rate (FDR) was used for parameter optimization during the feature selection. Long-term elevated serum bile acids were identified as risk factors for HCC development.

Metabolomics is increasingly applied to studies of pathogenesis and biomarker identification^1^,^2^. Metabolomics aims to comprehensively monitor alterations at the metabolic level in response to endogenous or exogenous stimuli^3^ and link metabolic disruptions to biological mechanisms^4^. Because metabolism is a dynamic process, it is very important to monitor the dynamic responses of metabolites in response to disease development and drug administration. Coupled with systematic metabolomics investigations, time-series^5^,^6^ studies are increasingly recognized as advantageous in disease pathogenesis research, early diagnosis, personalized medicine, and the elucidation of complex life processes.

Optional data processing methods for complex metabolomics time-course data are rare^6^. Most of algorithms were proposed for large sets of time-series data, while the number of time points in a metabolomics time-series study is often less than ten^7^. Short time series, together with large variables and small samples (characteristics of metabolomics data), render many classic data analysis methods unsuitable for metabolomics dynamic studies^6^,^8^.

Time-series data are frequently analyzed by static methods that do not consider their dynamic nature^6^. For example, three-dimensional data have been analyzed by means of PCA and PLS-DA, etc.^9^,^10^,^11^,^12^,^13^,^14^, without taking advantage of time information. Parallel factor analysis^15^ (PARAFAC) can resolve data with three or more dimensions and it can treat samples, features and time^16^ together to analyze overall metabolic trends. However, PARAFAC is a time-consuming process^17^, and the number of principal components chosen greatly influences the identification of physiologically relevant features^18^. Clustering algorithms are also applied to analyze time-series data^19^,^20^,^21^,^22^,^23^,^24^ to group the features according to their dynamic changes. Methods have been proposed to define important features by simulating the variable distribution or evaluating the smoothness of the variables at each time point^25^,^26^. To model short time series in metabolomics^25^, each observed time series is assumed to be a smooth random curve inferred by a functional data analysis approach. Berk et al.^7^ described a statistical framework for estimating time-varying metabolic data and used a functional test statistic to detect differences between groups. Trend analysis of time-series data^27^ is a method for untargeted metabolic feature discovery that employs two univariate methods: autocorrelation as a measure of the smoothness of non-random behavior and curve-fitting to analyze the compounds. Although these methods are compatible with short time-series datasets, each observed time series is assumed as a smooth random curve. However, when dealing with detailed time-series data where specific time points must be treated differently, corresponding data processing methods are needed.

Hepatocellular carcinoma (HCC) is one of the most lethal cancers^28^,^29^, and its incidence and mortality rates continue to increase^30^. However, the mechanism of hepatocarcinogenesis remains obscure because of the complicated interactions of multiple factors and individual genetic variations, impeding early clinical intervention before the development of HCC. Relatively effective treatments are available when HCC is diagnosed early. HCC patients often have a history of chronic liver diseases, leading to the introduction of screening programs among high-risk populations^31^, such as those infected with hepatitis virus B (HBV) in Qidong, China (a high-incidence area of HCC due to the high prevalence of HBV infection), who undergo HCC screening every half year. In addition, a sample library has been established in Qidong for HCC pathogenesis and early diagnosis studies^32^,^33^,^34^.

In this study, a weighted relative difference accumulation algorithm (wRDA) and its extended form were proposed. The wRDA method was first used to treat our previously published rat model data, and its extended form was further applied to a prospective cohort study of HCC patients with the aim of revealing earlier HCC diagnosis biomarkers and metabolic dysregulations contributing to hepatocarcinogenesis.

## Results

### The application of the wRDA to metabolomics data from the rat HCC model

The proposed wRDA was first applied to our previously published data for a rat HCC model induced by diethylnitrosamine (DEN) administration^35^. In that study^35^, 52 differential metabolites were identified, of which three, taurocholic acid (TCA), lysophosphoethanolamine 16:0 (LPE 16:0) and lysophosphatidylcholine 22:5 (LPC 22:5), were defined as “marker metabolites” for distinguishing the different stages of chemical hepatocarcinogenesis. LPE 16:0 and TCA were more discriminative between the disease group and control group, whereas LPC 22:5 was more discriminative between the HCC and non-HCC samples.

**Figure 1:**
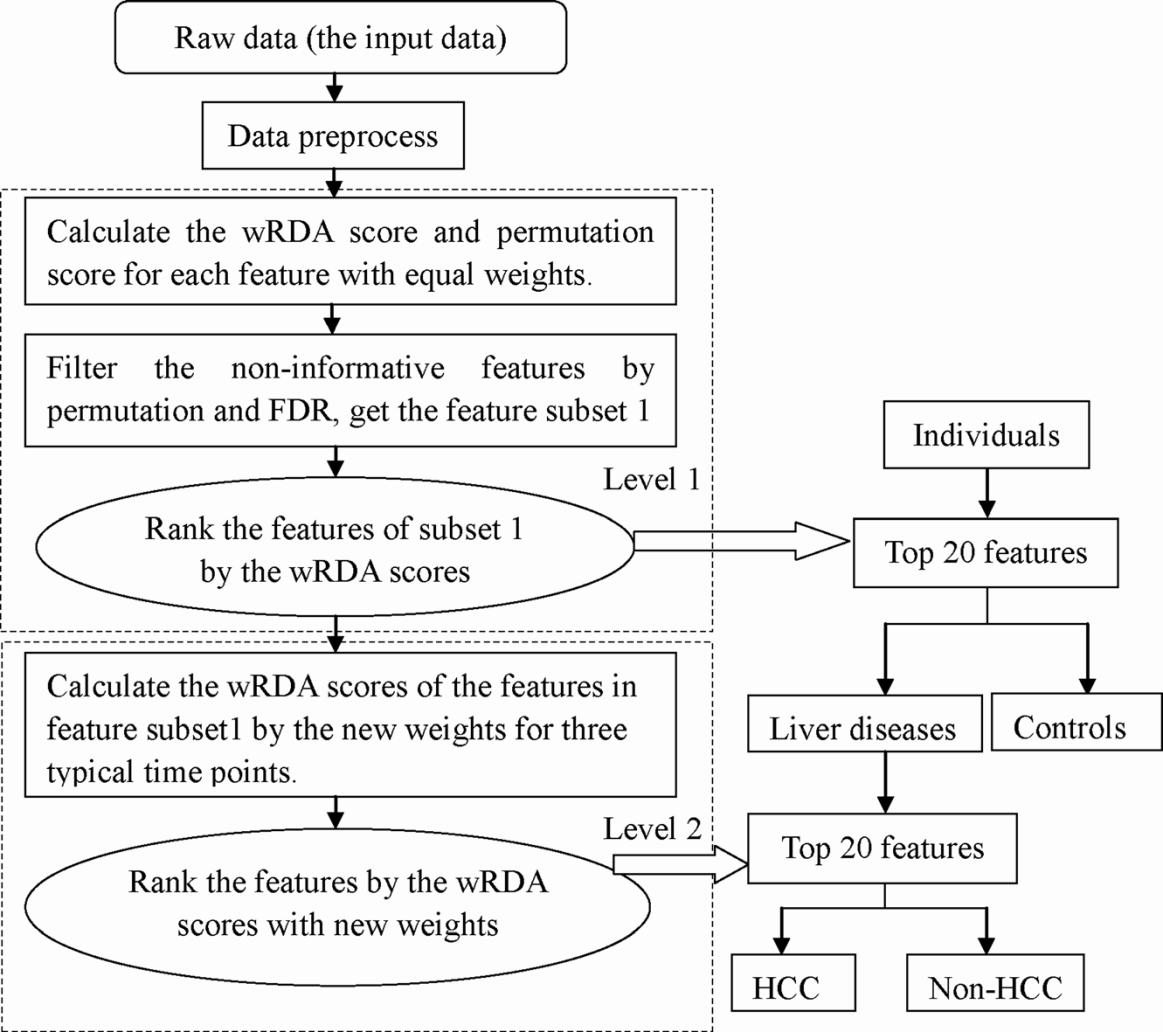
Flow chart of the analysis of the rat metabolomics data.

**Figure 2:**
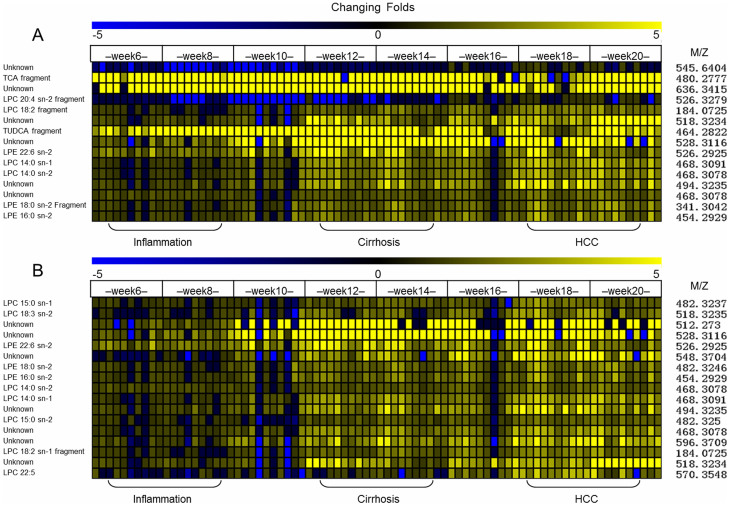
Top 20 features ranked by wRDA for discriminating liver diseases from healthy control (A) and HCC from non-HCC stages (B). Redundant ions from the same compound are deleted. LPC: lysophosphatidylcholine, TCA: taurocholic acid, TUDCA: tauroursodeoxycholic acid, LPE: lysophosphatidylethanolamine.

Parallel to our previous feature-defining process, a two-level data analysis procedure employing the wRDA (Fig. 1) was performed to select meaningful features to discriminate between the models and control, and between HCC and non-HCC samples. In the first level, 152 ion features were removed by means of permutation, leaving 1092 features with a false discovery rate (FDR) of 0 to constitute feature subset 1. Then, Support Vector Machine^36^ (SVM) was conducted based on the top 20 features (Fig. 2A) ranked by the wRDA. Five fold cross validation was conducted 50 times. The average accuracy rate was 98.85% ± 0.66%, demonstrating that the top ranked variables have a strong ability to distinguish disease samples from controls. These metabolic features include two bile acids (TCA and tauroursodeoxycholic acid (TUDCA)), LPCs, and LPEs with different acyl chains and unsaturation levels. These results indicate a disturbance of lipid metabolism in DEN-induced liver disease.

In the second level, the features in feature subset 1 were analyzed again to calculate their ability to characterize the metabolic status of the three different liver diseases. Using the top 20 ranked variables (Fig. 2B), the average accuracy rate of the SVM classifier for discriminating HCC and non-HCC samples was 93.12% ± 1.92%, demonstrating that informative features can be defined by weighting the time points according to their importance (here the importance of the time points was decided according to prior knowledge). The combinations of these features can denote disease progression towards HCC.

To investigate the discrimination abilities of the top ranked 20 features defined by wRDA to distinguish the liver diseases from the controls, their receiver operator characteristic curves are further drawn with their area under curves (AUCs) calculated. Among 15 features (after deleting the redundant ions from the same metabolite), three features TUDCA, feature with m/z of 636.3415 and LPE 22:6 are found to have higher AUCs of 0.97, 0.97 and 0.96, respectively (Fig. S1) than that of TCA (0.94). And 12 features have larger AUCs than that of LPE 16:0 (0.88) except for LPC 20:4 and feature with m/z of 545.6404 (0.81 and 0.79, respectively). TUDCA, feature with m/z of 636.3415 and LPE 22:6 were not included in 52 differential metabolites defined by VIP > 2 in the analysis of PLS-DA in our previous work^35^. The Pearson correlation coefficients of TUDCA and feature with m/z of 636.3415 with TCA are 0.71 and 0.94, respectively. LPE 22:6 also has a high Pearson correlation coefficient with LPE 16:0 (0.80). For discriminating HCC from non-HCC, LPC 22:5 has the largest AUC (0.87). Because the top ranked features are related to bile acid and lipid metabolism disturbance which was discussed in our previous paper^35^, here further explanation is omitted. Collectively, the metabolic features derived from the rat model metabolomics data demonstrate the excellent performance of the wRDA in feature-ranking and demonstrate its potential in analyzing time-series metabolomics data.

### The application of w^2^RDA in HCC prospective metabolomics data analysis

In this prospective cohort study, samples of 5 time points from 11 HCC cases were collected during screening over 3.5 years. The study aimed to identify prospective features of HCC, but the biological events in the other time points before HCC occurrence are unknown. Moreover, in contrast to animal model samples, the collection times of samples from patients in the same stage were not uniform. Therefore, parameter *k* was introduced into the wRDA to reduce the influence of sample storage time. The settings of *ω_i_* for each time point and *k_j_* for each sampling time affect the measurement of the features. To define the most suitable settings for *ω* and *k*, linear function, exponential function and proportional function were tested with different changing factors (equal weights were included as a special case of linear function). For *n* top-ranked features, the lowest FDR was adopted to evaluate the settings of *k* and *ω*. As shown in Table 1, when *ω* was an exponential function, the minimum values of the lowest FDR were obtained for each *k'*s function setting. When *k* was also an exponential function, the minimum FDR values in each column were derived for the vast majority of different function settings of *ω* for *n* decreasing from 50 to 30. Furthermore, 194 *k*-*ω* pairs were derived from different functions and their corresponding changing factors, resulting in a low FDR of less than 5% (including 192 *k*-*ω* pairs with the lowest FDR equaling 0 at *n* = 30 and 2 *k*-*ω* pairs with the lowest FDR equaling 2.86% at *n* = 35). Among the 194 *k*-*ω* pairs, the probability of both *ω* and *k* being exponential was the highest (25.26%). Therefore, exponential function is more suitable for *k* and *ω* than other functions.

When *k* and *ω* were fixed as exponential functions, the lowest FDR was 0% at *n* = 30, and there were 47 paired values of changing factors for *k* and *ω* (Table S1). To minimize the weight differences among the sampling points, the smallest changing factor of *k* was chosen, *q_k_* = 0.5. Once *k* was selected, the smallest changing factor of *ω* was defined as *q_ω_* = 0.6.

Under the optimized settings for *k* and *ω*, the features were ranked according to their w^2^RDA scores. The top 30 ranked variables (Table 2) were chosen as the most important metabolic features related to HCC development.

**Figure 3:**
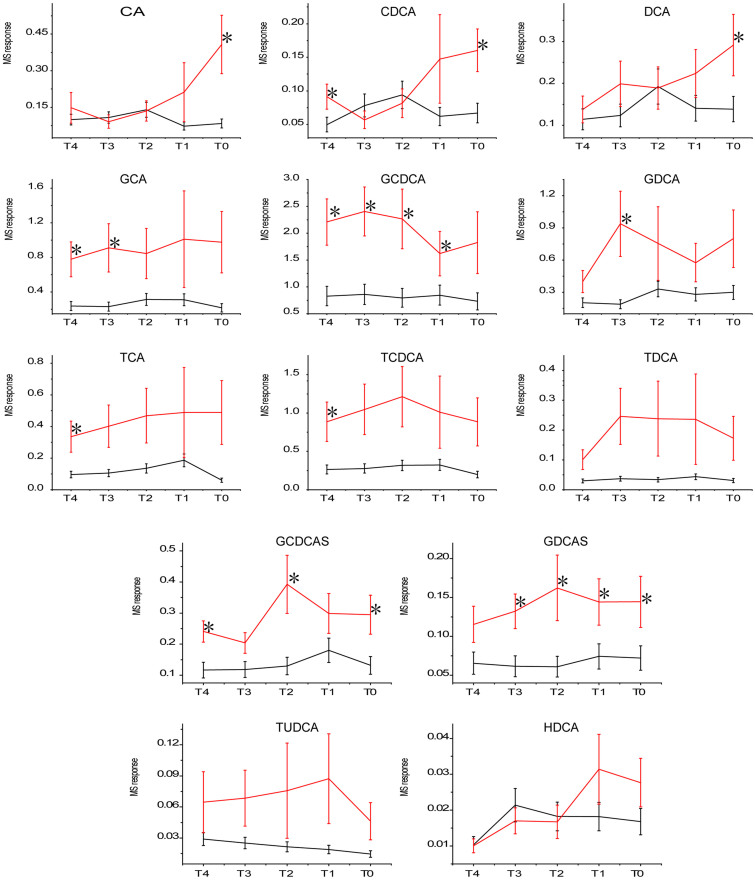
Relative content of serum bile acids in the HCC group compared to the HBsAg^+^ control group at paired time points. T_0_: the stage at which patients were initially diagnosed with HCC. Serum samples collected every 6 months prior to T_0_ at T_1_ (half a year ago), T_2_ (one year ago), T_3_ (one and a half years ago), T_4_ (two years ago) were then identified. Abbreviations are the same as in Table 2. * indicates significance (*p* < 0.05).

The most important variables were bile acids, with the exception of dihydroxyandrostenone sulfate and LPE 18:2. The serum concentrations of the primary bile acids cholic acid (CA) and chenodeoxycholic acid (CDCA) were significantly higher in the HCC group than in the control group (at T_0_) when malignant hepatic tumors were identified. Four other bile acids, the secondary bile acids deoxycholic acid (DCA) and taurodeoxycholic acid (TDCA) and the sulfated bile acids glycodeoxycholate sulfate (GDCAS) and glycochenodeoxycholate sulfate (GCDCAS), were also elevated in HCC sera compared to controls. When the serum levels of bile acids were compared over the entire monitoring time period, most were significantly elevated in patients in which HCC occurred compared to individuals that were hepatitis B surface antigen positive (HBsAg^+^), with the exception of hyodeoxycholic acid (HDCA) (Table 2). The relative serum levels of bile acids at each time point are shown in Fig. 3.

## Discussion

HCC usually develops from chronic liver diseases, and a long time is required for the formation of malignant hepatic tumors. The mechanism underlying the occurrence and development of HCC remains to be elucidated.

Bile acids are synthesized in the liver and their functions are not limited to facilitating the absorption of lipids and lipid-soluble nutrients^37^,^38^ but also include acting as signaling molecules to regulate glucose and lipid metabolism^39^,^40^ and apoptosis^41^. Bile acids are detergents and are cytotoxic, and their concentrations are tightly regulated under normal physiological conditions^42^,^43^. We previously demonstrated that glycocholic acid (GCA) and glycochenodeoxycholic acid (GCDCA) are elevated in hepatitis, cirrhosis and HCC accompanied by cirrhosis^44^. The serum levels of seven bile acids were quantitatively measured and compared among HCC patients without liver cirrhosis and hepatitis, HCC patients with liver cirrhosis and hepatitis, benign liver tumor patients with liver cirrhosis and hepatitis, and healthy controls, and elevated serum GCDCA, GCA and TCA levels and decreased serum CDCA levels were correlated with liver cirrhosis and hepatitis^45^. We previously demonstrated that conjugated GCA, GCDCA, TCA and taurochenodeoxycholic acid (TCDCA) are potential biomarkers of liver cirrhosis^46^.

Fasting serum levels of primary bile acids^47^ can be affected by enterohepatic circulation^48^, leading to intra-individual variations. Thus, multiple time points of circulating bile acids were compared together within the monitoring period instead of at a single time point. This comparison revealed that all bile acids except HDCA were significantly higher in HCC patients than in HBsAg^+^ controls. The more hydrophobic secondary bile acids DCA and lithocholic acid (LCA) have been reported to increase HCC risk^49^,^50^.

Activation of the YAP pathway was recently shown to be responsible for bile acid-dependent tumor promotion^51^. The development of spontaneous liver tumors in a Fxr^-/-^ Shp^-/-^ double-knockout (DKO) mouse model was employed in the study to produce chronically elevated bile acid levels, which enabled the study of the mechanism of hepatic malignant tumor promotion by long-term high circulating levels of the bile acids CA and CDCA in mice^51^. Compared to the HBsAg^+^ control group, serum CA, CDCA and DCA levels were slightly elevated in the HCC group during the two-year monitoring period, and their glycine- and taurine-conjugated forms were elevated to a greater extent. Thus, the increased serum levels of bile acids may be due to leakage from damaged hepatic cells or the alteration of bile acid transfer protein activity^52^ rather than upregulation of bile acid synthesis. When more bile acids enter the blood, they may further intensify hepatic injury because of their cytotoxic nature and may simultaneously act as signaling molecules to promote hepatic tumor formation.

No differences in levels of ursodeoxycholic acid (UDCA), which has been reported to have protective effects^53^,^54^, were observed in HCC patients and HBsAg^+^ controls during the two years before or at HCC diagnosis, whereas levels of its taurine-conjugated form were slightly elevated in HCC patients. The sulfated bile acids GDCAS and GCDCAS were elevated in HCC patients during the two years prior to diagnosis. Sulfotransferase-2A1, which has been reported to be underexpressed in HCC tumor cells^55^, catalyzes the sulfation of bile acids for their elimination and detoxification^56^. Sulfotransferase activities have been reported to decrease with the severity of liver disease from steatosis to cirrhosis^57^. The long-term increase in sulfated bile acids in HCC patients may be due to their increased availability for sulfation rather than enhanced SULT2A1 activity.

Because chronic hepatitis and cirrhosis are typically precursors of HCC, in combination with the above evidence that bile acids promote hepatic tumor formation, it is reasonable to speculate that long-term high circulating bile acids are potential high-risk factors for HCC. TCA is elevated since week 6 in model rats treated with DEN compared to controls^35^. The 13 bile acids mentioned above were extracted from the DEN-induced rat HCC model data acquired in positive mode, which revealed that the 10 bile acids (CA, CDCA, DCA, GCA, GCDCA, glycodeoxycholic acid (GDCA), TCA, TCDCA, TDCA and tauroursodeoxycholic acid (TUDCA)) detected were elevated in sera in the model group compared to the control group since week 6 (Supplementary Fig. S2), coincident with the appearance of hepatic cell injury due to DEN treatment. It has been reported that 40% of HCC patients infected with HBV from Qidong have high exposures to aflatoxin B1^58^. Although the mechanism of HCC pathogenesis may vary greatly because of different etiological agents, the common long-term elevated serum bile acids were observed before HCC occurrence. Collectively, it can be speculated that the elevated levels of circulating bile acids in chronic liver disease may play an important role in the process of malignant hepatic tumor formation in both humans infected with HBV and DEN-treated rats.

In this article, to identify discriminative metabolites that may reflect dynamic biochemical developments, a wRDA method based on the weighted mean and variance analysis along the time points was proposed. Rather than screening differentially expressed variables at isolated time points as in static methods, the wRDA can investigate variables comprehensively along all time points in feature selection. Moreover, weighting the time points emphasizes the influence of relevant, important time points by setting relatively larger corresponding weights, and vice versa. Thus, high efficiency can be achieved in identifying the key differences between two groups along the entire time course.

The application of the wRDA to the rat metabolomics data demonstrated that it is an effective method for defining metabolic features that may be related to disease status. In the prospective cohort study, continuously high serum bile acid levels within a two-year monitoring time period were identified as risk factors for HCC development. The weights of the time points can be decided based on prior knowledge or optimized by the lowest FDR. Other functions and changing factors for weighting can be simulated, and other optimization standards can be introduced in further studies. Collectively, our proposed method, by analyzing the weighted relative difference accumulation along the time dimension, effectively defines the features of dynamic metabolism related to disease development.

## Methods

### wRDA and its application to the rat liver disease model

#### wRDA

When analyzing the metabolomics time course data of two different groups, for simplicity, let C denote the control group and M denote the model group. Let *T_i_* denote a time point, 0 ≤ *i* < *N*, where *N* is the number of the time points.

In bioinformatic data analysis, the method used to measure the discriminative ability of a feature among different groups is a key consideration. SAM^59^ scores the “relative difference” of a gene over repeated measurements according to the mean and the standard deviation. Based on the idea of “relative difference”, the wRDA considers the variations of the means along the time points to dynamically study the biological process and screen biomarkers that reflect differences between the two groups and characterize the development of the model group. A variable with a higher wRDA score is more discriminative between the two groups. The detailed principles of the wRDA are outlined as follows:

In metabolomics time-series studies, metabolites are measured at each time point. Because differences in metabolite levels in the two groups may occur in a process, the accumulation of distance between the mean values of a given feature *f* at all time points, *D*(*f*), reflects the discriminative ability of feature *f*:
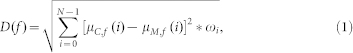
where *μ_C_*_,*f*_(*i*) and *μ_M_*_,*f*_(*i*) are the mean values of feature *f* at time point *i* in the C and M groups, respectively and *ω_i_* represents the weight of time point *i*. Different time points may play different roles in the development of differences between the two groups, resulting in different weight settings. In particular, some time points may occur at typical stages for biological events and hence merit greater attention and relatively large weights.

Furthermore, the standard deviation is applied to enable a fair comparison among the discriminative abilities of features. Let
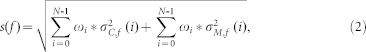
where σ*_C_*_,*f*_(*i*) and σ*_M_*_,*f*_(*i*) are the standard deviations of feature *f* at time point *i* in the C and M groups, respectively. Hence the “weighted relative difference accumulation” of a feature *f* between the C and M groups over all time points is calculated as *wRDA*(*f*):
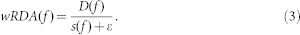
*ε* is a small positive value introduced to moderate or regularize the wRDA score and potentially reduces the relative impact of small variances. In this study, *ε* was set to 0.005.

#### Data source for the rat liver disease model

Metabolomics dynamic data for the rat liver disease model^35^ were employed using the model obtained from the Shanghai Experimental Animal Centre. Serum samples were collected from two groups, control rats and rats with liver disease induced by DEN, every two weeks from week 6 to week 20. A total of 8 monitoring time points were obtained. In this animal model, the serial progression of hepatocarcinogenesis includes three disease stages: the inflammation stage (week 6–week 8), the cirrhosis stage (week 12–week 14), and the HCC stage (week 18–week 20). All samples at each time point were collected synchronously in this animal experiment.

#### The application of the wRDA to the rat liver disease model

The wRDA was first applied to the metabolomics data of the rat liver disease model. First, features whose values equaled zero in more than 20% of the samples^60^ at each time point were removed, leaving 1289 features. Then, outlier correction was conducted (a sample for feature *f* is an outlier if its value is beyond the range *μ*(*f*) ± 2σ(*f*), where *μ*(*f*) is the mean value of *f* and σ(*f*) is the standard deviation of *f* in the corresponding group^13^). Assuming that the feature followed a prior distribution, the outlier was replaced by a random selected sample value following this distribution.

To select the features reflecting the different developments between the two groups and define the features discriminating HCC samples from non-HCC samples, the wRDA was applied at two levels (Fig. 1).

(1) In the first level, to study the dynamic differences between the liver disease group and the control group, equal values of 1/8 were assigned to *ω* at all time points. The features defined together reflect the entire liver disease status of the model group from week 6 to week 20. Permutation was conducted 200 times to filter noise and non-informative features.

(2) At the second level, the wRDA was applied again to measure the variables in feature subset 1 (Fig. 1), and the weights (*ω*) of three time points, week 6, week 12 and week 18, which were defined as the typical stages of hepatitis, cirrhosis and liver cancer, were set to 0.3, 0.3, and 0.4, respectively. The stage at which liver malignant tumors occur is the most important and merits greater attention, as reflected by a larger weight. The weights (*ω*) of the other time points were set to zero. The weight adjustment allowed the most informative features characterizing the three different liver diseases to be targeted, particularly those capable of distinguishing HCC from non-HCC.

### w^2^RDA and its application in the HCC prospective cohort study

#### w^2^RDA

In epidemiological screening, people with high risk are checked at certain time intervals. One time point may lie in the onset stage of a disease or a malignant tumor, and other time points may be a certain time interval before or after the key biological event. However, not all patients with a disease or a malignant tumor were spot at a uniform screening period, and thus samples at each disease stage may be collected at different sampling times. To measure the features more accurately, a weight *k* for different sampling times was introduced:
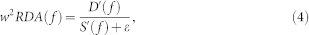

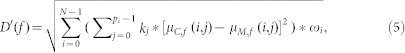
where *p_i_* is the number of the sampling times at time point *i*; *μ_C_*_,*f*_(*i*, *j*) and *μ_M_*_,*f*_(*i*, *j*) are the average levels of feature *f* at the sampling time *j* of the *i*^th^ time point in the C and M groups, respectively; and *k_j_* is the weight of the *j*^th^ sampling time. The extended standard deviation by considering the sampling time differences at each time point is defined as follows:
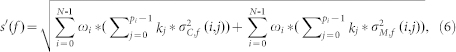
where σ_C,*f*_(*i*.*j*) and σ_M,*f*_(*i*,*j*) are the standard deviations of feature *f* at the sampling time *j* of the *i*^th^ time point in the C and M groups, respectively.

#### HCC prospective cohort study and data collection

Serum specimens were obtained from a prospective cohort in Qidong, Jiangsu Province, China. From May 2009 to October 2012, residents of the Qidong area were invited for a health examination as part of the HCC screening study. Table 3 shows the baseline characteristics of the enrolled HCC and HbsAg^+^ control subjects. Each participant underwent serological hepatitis tests (HBsAg, hepatitis B e antigen (HBeAg), anti-hepatitis C virus (HCV)), abdominal ultrasonography (US), serum alpha-fetoprotein (AFP) and alanine aminotransferase (ALT) tests at approximately six-month intervals. If an individual had abnormal results from US or higher AFP levels (greater than 20 ng/mL), then intensive surveillance by computed tomography (CT), magnetic resonance imaging (MRI), and/or hepatic angiography were employed to identify the space-occupying lesion. In total, 11 HCC cases were identified in a 3.5-year screening period. Their serum samples at the time of HCC diagnosis and samples within the 2 years preceding the initial HCC diagnosis were selected for analysis. Another 22 HBsAg^+^ individuals were taken as a positive controls with matched age, sex, sample collection time points and storage conditions (−20°C). The details of serum sample preparation, liquid chromatography-mass spectrometry (LC-MS)-based metabolic profiling and data preprocessing are available in the supplementary material.

#### The application of the w^2^RDA in the HCC prospective cohort study

**Figure 4:**
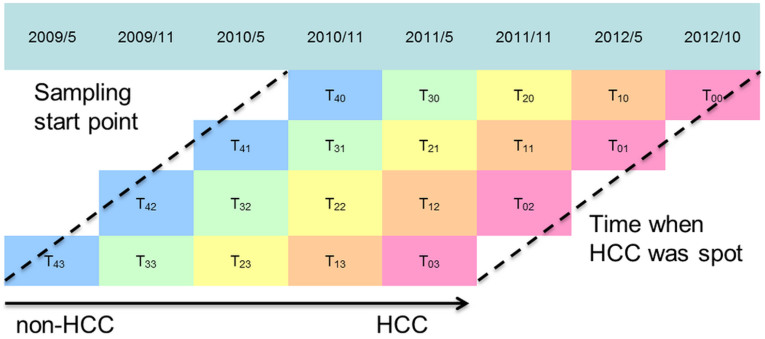
Sampling for HCC and HBsAg^+^ control groups from May 2009 to October 2012. T_0_: the stage at which patients were initially diagnosed with HCC. Serum samples were collected every 6 months prior to T_0_ at T_1j_ (half a year ago), T_2j_ (one year ago), T_3j_ (one and a half years ago), and T_4j_ (two years ago).

For simplicity, the HCC and HBsAg^+^ control groups are also denoted as M and C, respectively. T_0j_, T_1j_, T_2j_, T_3j_ and T_4j_ (Fig. 4) are used to mark each stage (time points). T_0j_ represents the stage when HCC was diagnosed, whereas T_1j_, T_2j_, T_3j_ and T_4j_ represent the stages 0.5, 1, 1.5, and 2 years before T_0j_, respectively. HCC patients were identified from the participants at four screening periods in this study; thus there are four sampling times for each stage (Fig. 4). Taking the T_0j_ stage as an example, patients were diagnosed with liver cancer in May 2011, Nov. 2011, May 2012 and Oct. 2012. The corresponding serum samples at 6 months, 12 months, 18 months and 24 months prior to HCC diagnosis were then collected. The longest time interval of the samples at each stage from different sampling times was 1.5 years. Therefore, the w^2^RDA was applied to further consider the possible influence of different sampling times.

The aim of the Qidong cohort study was to identify prospective features related to the occurrence of HCC. The settings of the weights *ω* and *k* could affect the feature measurement of w^2^RDA. T_0j_ is the onset stage of HCC and therefore is the most important. The closer the other time points are to T_0j_, the more similar their metabolic characteristics are to those of HCC. Hence, for *ω* (and *k*), three different setting methods were tested: a linear function, a proportional function and an exponential function. FDR^59^,^61^ was adopted to evaluate the results under different weight settings. For the linear function *ω_i_* = 1.0 + (*N*-*i-*1)*q*, 0 ≤ *i* < *N*, *q* is a parameter factor. When *q* = 0, all time points have the same weight. For the proportional function *ω_i_* = (1.0 + *q*)^(*N*-*i-*1)^, 0 ≤ *i* < *N*. Many metabolomics experiments use natural exponential functions to estimate time-varying profiles^6^; in this case, *ω_i_* = *e* ^(*N-i−*1)*q*^, 0 ≤ *i* < *N*. In the latter two functions, *q* is also a changing factor. In the Qidong cohort study, to consider the influences of all the monitoring time points on metabolism, the differences of *ω_i_* should not be too large. *q* was restricted from 0.1 to 1.0 and was tested with a step increment of 0.1. In addition, T_0j_ is the most important stage when malignant hepatic tumors are discovered, and thus its weight should be larger than those of the others. Similarly, the weight *k* was also tested using a linear function, proportional function and exponential function. The sample with the shortest storage time should have the greatest weight.

## Author Contributions

W.Z., L.Z. and P.Y. researched data, wrote the manuscript; J.W., J.C., X.L. and X.W. contributed to the clinical sample organization and discussion; X.L. and G.X. contributed to discussion, reviewed/edited the manuscript.

## Supplementary Material

Supplementary Information Supplementary materials

## Figures and Tables

**Table 1:** Optimization of *k* and *ω* according to the lowest FDR values under different function settings

*n*the lowest FDR value*k*'*s*, ω'*s functions*	50	45	40	35	30
*a, a′*	44.00	35.56	35.00	40.00	36.67
*a, b′*	36.00	35.56	37.50	31.43	30.00
*a, c′*	34.00	33.33	35.00	31.43	20.00
*a, d′*	34.00	35.56	35.00	31.43	30.00
*b, a′*	40.00	37.78	40.00	31.43	23.33
*b, b′*	36.00	35.56	40.00	22.86	10.00
*b, c′*	34.00	35.56	32.50	17.14	3.33
*b, d′*	34.00	35.56	32.50	20.00	6.67
*c, a′*	40.00	37.78	37.50	20.00	0.00
*c, b′*	34.00	35.56	30.00	20.00	0.00
*c, c′*	34.00	33.33	30.00	2.86	0.00
*c, d′*	34.00	33.33	30.00	14.29	0.00
*d, a′*	40.00	37.78	37.50	20.00	6.67
*d, b′*	34.00	37.78	32.50	22.85	0.00
*d, c′*	34.00	33.33	32.50	17.14	0.00
*d, d′*	34.00	35.56	32.50	20.00	0.00

*Combinations of *k* and *ω* for four different functions were tested; the lowest derived FDR values are listed. Functions for *k* and *ω*: *a* and *a′*-equal weights; *b* and *b′*-linear function; *c* and *c′*-exponential function; *d* and *d′*- proportional function.

**Table 2:** Top 30 variables ranked by the w^2^RDA in the HCC prospective study

							*p* value	
Ranking No.	Score	t_R_	m/z	Compounds	Existing form	Ion mode	M_0_ vs C_0_	M vs C
1	1.17	9.95	528.2631	GDCAS	M−H	ESI−	0.02	4.47E−06
2	1.12	11.73	448.3063	GCDCA	M−H	ESI−	0.09	5.29E−07
3	1.07	10.32	498.2889	TCDCA	M−H	ESI−	0.05	2.80E−05
4	1.07	10.65	498.2889	TDCA	M−H	ESI−	0.08	7.19E−04
5	1.05	10.13	464.3012	GCA	M−H	ESI−	0.06	1.50E−04
6	1.01	9.05	514.2838	TCA	M−H	ESI−	0.06	4.02E−04
7	1.01	11.57	407.2791	CA	M−H	ESI−	0.02	2.14E−02
8	0.99	10.67	500.3031	TDCA	M+H	ESI+	0.04	1.14E−03
9	0.99	9.68	528.2631	GCDCAS	M−H	ESI−	0.03	6.13E−06
10	0.98	12.07	448.3063	GDCA	M−H	ESI−	0.1	4.79E−04
11	0.98	13.98	391.2848	DCA	M−H	ESI−	0.04	2.02E−02
12	0.96	19.99	802.5942	UN	—	ESI+	0.57	3.06E−01
13	0.93	8.9	498.2889	TUDCA	M−H	ESI−	0.11	4.16E−03
14	0.92	9.73	432.3106	GCDCAS	Fragment	ESI+	0.05	2.87E−05
15	0.9	12.1	450.3206	GDCA	M+H	ESI+	0.13	2.11E−04
16	0.89	13.68	391.2848	CDCA	M−H	ESI−	0.02	3.28E−02
17	0.88	10.09	929.6078	GCA	2M−H	ESI−	0.13	1.36E−02
18	0.87	7.41	383.1522	DHEAS	M−H	ESI−	0.02	2.61E−06
19	0.87	19.79	228.1955	UN	—	ESI+	0.34	1.85E−01
20	0.87	20.3	802.5941	UN	—	ESI+	0.92	2.56E−01
21	0.86	17.5	524.3701	UN	—	ESI+	0.79	5.75E−01
22	0.86	10.32	500.3046	TCDCA	M+H	ESI+	0.21	1.35E−03
23	0.86	12.45	391.2848	HDCA	M−H	ESI−	0.16	2.66E−01
24	0.85	14.36	476.2763	LPE 18:2 sn-2	M−H	ESI−	0.13	4.80E−05
25	0.83	12.04	897.6172	GDCA	2M−H	ESI−	0.18	1.68E−02
26	0.82	10.31	999.6003	TCDCA	2M+H	ESI+	0.13	3.83E−03
27	0.8	11.54	373.2736	CA	Fragment	ESI+	0.03	2.10E−02
28	0.79	9.93	931.6241	GCA	2M+H	ESI+	0.18	2.52E−02
29	0.78	17.25	524.3702	UN	—	ESI+	0.58	8.13E−01
30	0.78	9.75	450.3218	GCDCAS	Fragment	ESI+	0.07	4.80E−02

M represents the group of patients who were diagnosed as having HCC, and C means HBsAg^+^ control group. M_0_ and C_0_ represent the HCC group and control group at time point T_0_. CA: cholic acid, CDCA: chenodeoxycholic acid, DCA: deoxycholic acid, GCA: glycocholic acid, GCDCA: glycochenodeoxycholic acid, GDCA: glycodeoxycholic acid, TCA: taurocholic acid, TCDCA: taurochenodesoxycholic acid, TDCA: taurodeoxycholic acid, GCDCAS: glycochenodeoxycholate sulfate, GDCAS: glycodeoxycholate sulfate, HDCA: hyodeoxycholic acid, TUDCA: tauroursodeoxycholic acid, UN: unknown compounds or ions, DHEAS: 3b,16a-Dihydroxyandrostenone sulfate, LPE: lysophosphatidylethanolamine.

**Table 3:** Baseline characteristics of the enrolled HCC and HBsAg^+^ control subjects at T_0_

	HCC patients (*n* = 11)	Controls (*n* = 22)
Age (year, range, median)	46–64, 52	44–66, 54
Sex (male/female)	9/2	18/4
HBsAg positive (Number)	11	22
ALT > 45 (U/L)	—	—
AFP > 20 (ng/mL)	6	1

HBsAg, hepatitis B surface antigen; ALT, alanine aminotransferase; AFP, alpha-fetoprotein.
