# Supplementary material for: A weighted relative difference accumulation algorithm for dynamic metabolomics data: long-term elevated bile acids are risk factors for hepatocellular carcinoma
Source: Sci Rep. 2015 Mar 11;5:8984. doi: 10.1038/srep08984 (PMC4355672; doi:10.1038/srep08984)
Supplement: Supplementary Information — Supplementary materials [file srep08984-s1.doc]

**Supplementary materials**

**A weighted relative difference accumulation algorithm for dynamic metabolomics data: long-term elevated bile acids are risk factors for hepatocellular carcinoma**

Weijian Zhang^1‡^, Lina Zhou^2‡^, Peiyuan Yin^2^, Jinbing Wang^3^, Xin Lu^2^, Xiaomei Wang^1^, Jianguo Chen^3^, Xiaohui Lin^1^* & Guowang Xu^2^

^1^School of Computer Science & Technology, Dalian University of Technology, Dalian, China

^2^Key Laboratory of Separation Science for Analytical Chemistry, Dalian Institute of Chemical Physics, Chinese Academy of Sciences, Dalian, China

^3^Qidong Liver Cancer Institute, Qidong 226200, China.

^‡^Two authors contributed equally

**Address for Correspondence:**

*Xiaohui Lin, School of Computer Science & Technology, Dalian University of Technology, 2 Linggong Road, Dalian 116023, China. Tel.: +86-411-84706002 ext.3920, Fax: +86-411-84379559, e-mail: datas@dlut.edu.cn.

**Metabolic profiling experimental for HCC prospective cohort study**

**Chemicals** Acetonitrile (HPLC-grade) (Merck, Darmstadt, Germany), formic acid (Sigma- Aldrich, St. Louis, MO, USA), laboratory-prepared Milli-Q water (Millipore, Bedford, MA) were used to prepare mobile phases. Chemical standards used as internal standard and compound characterization were purchased from Sigma-Aldrich (St. Louis, MO, USA).

**Sample preparation** For each sample, 100 μL serum was drawn and four volumes of acetonitrile (with internal standard mixture of carnitine C2:0-d3, carnitine C10:0-d3, carnitine C16:0-d3, LPC 12:0, LPC 19:0, FFA 16:0-d3, FFA 18:0-d3, chenodeoxycholic acid-d4, cholic acid-d4, tryptophan-d5, phenylalanine-d5 and lansoprazole.) were added for protein precipitation. Vortex and centrifugation (15,000 x g for 10 min at 4 °C) were followed. Then the supernatant was dried in a vacuum centrifuge and stored at -80 °C before LC-MS analysis. For reconstitution, 100 μL acetonitrile/water (1:4, v/v) was used. After vortex and centrifugation (15,000 x g for 10 min at 4 °C), supernatant was used for injection. By pooling equal aliquots of plasma from real samples, quality control (QC) samples containing average metabolite information of real samples were pretreated as the above real samples. The QC sample was run every 10 real samples to evaluate the repeatability of sample pretreatment and the LC-MS batch analysis.

**LC-MS-based metabolic profiling** For metabolic profiling, an ultra high performance liquid chromatograph (UPLC) (Waters, USA) was coupled to a LTQ Orbitrap XL hybrid mass spectrometer system (Thermo Fisher, USA) equipped with an electrospray source. The injection volume was 5 μL with the injection chamber temperature controlled at 10 °C. During chromatographic separation, a 100 mm HSS T3 column (Waters, Milford, MA, USA) was used with inner diameter of 2.1 mm and particle size of 1.7 µm. The gradient started with 5% B (acetonitrile), maintained for 1 min, linearly increased to 100% B at 22 min and held for 3 min. The total run time for each injection was 30 min, including a four-minute post-equilibration with 95% A (0.1% formic acid in water). The constant column temperature and flow rate were set at 50 °C and 0.35 mL/min. During MS signal acquisition, the scan range was set as 50 - 500 daltons for the first 2 min segment and 100 - 1100 daltons for the following segment. The resolution was set as 30 K. The ion source parameters set for profiling were referred to our previous settings[^1^](#_ENREF_1). The data were stored in profiling format.

**Data preprocessing** Peak detection and matching were performed using SIEVE software (V2.1, Thermo Fisher). The target ranges of m/z and retention time were set as 50-1000 Dalton and 0.01-26 min. For framing, the retention time width (the combination of the peak width and drift window) and mass width (drift window for mass accuracy) were set as 0.5 min and 30 ppm; maximum frame number and peak threshold were set as 5000 and 50000, respectively. Other parameters were set as default. Then a large matrix of integrated peak areas with variables (m/z, retention time) in rows and samples in the columns was exported to an Excel table for both positive and negative modes, respectively. Ion fusion of fragmentation ions, adduct ions and isotopes from a common molecule were firstly exerted according to our previous procedure[^2^](#_ENREF_2). Isotope distribution pattern, adduct ions and neutral loss rules were used in combination, correlation coefficients not smaller than 0.6 is considered for each case, as well. The parameters for ion fusion were as follows with some modifications: the window for mass was within ±0.005 Da, the window for retention time was within ±0.05 min. The independent correlation coefficients were set as not smaller than 0.95 to minimize false positives. The ions with the most abundance in each fusion group were kept. The variables in the fused peak table were deleted if their values equal to zeros in more than 20% samples in each group[^3^](#_ENREF_3). Each variable was normalized to multiple internal standards and the corresponding RSDs of all QC samples were calculated, the best internal standard was the one that results in a minimal RSD[^4^](#_ENREF_4).

**Permutation** Metabolomics data are often high dimensional and usually contain noise and non-related variables, thus permutation and artificial variables[^5^](#_ENREF_5) are adopted to filter the non-informative data. In permutation, the values are randomly rearranged for each feature *f* among the samples of the two groups at the same time point and a new man-made feature, *mf*, is constructed. Then wRDA method are performed on the *mf* data. Permutations are done for *t*>1 times for each *f* to reduce the randomness and an average permutation score for each *mf* is obtained. If *avr-wRAD*(*mf*)≥*wRAD*(*f*) (or *avr-w*^2^*RAD*(*mf*)≥*w*^2^*RAD*(*f*)), then *f* is non-informative and will be removed from the feature set. That is, the information contained in the feature may be distorted by randomly rearranging the values of the feature, and the wRDA or w^2^RDA score of its *mf* will decrease correspondingly.

**The false discovery rate** False discovery rate (FDR)[^6^](#_ENREF_6)^,7^ is an effective tool to evaluate the selected feature subset.

Features are ranked according to their wRDA (or w^2^RDA) scores in a descending order. The top ranked features are the most informative ones. and the top *n* ranked features could be defined or the features with scores greater than α could be selected with α as a cut-off score. Different parameter settings could induce the changes of feature rankings, resulting in different feature subsets. To evaluate the influences of the parameters, FDR[^6^](#_ENREF_7)^,7^ is adopted. To calculate FDR, permutation is also conducted. Assume *n* is the number of variables to be selected, and the score of the *n*th feature is α. Let *n*_1_ be the number of the variables whose average permutation scores are greater than α, then FDR is *n*_1_/*n*. The lower the FDR is, the better the selected features are.

**Statistical analysis after feature selection.** Heatmap was exhibited employing the software of Multi Experiment Viewer ([http://www.tm4.org](http://www.tm4.org/)) providing the relative abundances of top ranked features in model rats compared to control rats. Student’ s *t* test analysis was performed employing SPSS 13.0 (SPSS, Chicago, IL) to compare the relative levels of feature ions or target metabolites. The significant level was set as *p* <0.05.

**Structural identification of important features.** The detailed structural identificaton process of target ions are according to our previous procedure[^8^](#_ENREF_38). Their quasi-molecular ions should be firstly defined according to the isotope distributions, adduct ions, etc. And then the possible compounds are searched in the online databases (METLIN (http://metlin.scripps.edu/), HMDB (http://www.hmdb.ca/), Chemspider (http://www.chemspider.com/), lipidmaps (<http://www.lipidmaps.org/)>) with the accurate mass smaller than 3 ppm. Also, the MS2 fragments and the retention times are used for function group deducements. MS2 information and LC-MS retention behaviours of chemical standards are analyzed for the final validations.

Reference list

1. Zhou, L. *et al.* Metabolic profiling study of early and late recurrence of hepatocellular carcinoma based on liquid chromatography-mass spectrometry. *J. Chromatogr. B* **966**, 163-170 (2014).

2. Zeng, Z.D. *et al.* Ion Fusion of High-Resolution LC MS-Based Metabolomics Data to Discover More Reliable Biomarkers. *Anal. Chem.* **86**, 3793-3800 (2014).

3. Smilde, A.K., van der Werf, M.J., Bijlsma, S., van der Werff-van-der Vat, B.J.C. & Jellema, R.H. Fusion of mass spectrometry-based metabolomics data. *Anal. Chem.* **77**, 6729-6736 (2005).

4. van der Kloet, F.M., Bobeldijk, I., Verheij, E.R. & Jellema, R.H. Analytical Error Reduction Using Single Point Calibration for Accurate and Precise Metabolomic Phenotyping. *J. proteome res.* **8**, 5132-5141 (2009).

5. Lin, X. *et al.* A support vector machine-recursive feature elimination feature selection method based on artificial contrast variables and mutual information. *J. Chromatogr. B Analyt. Technol. Biomed. Life Sci.* **910**, 149-155 (2012).

6. Benjamini, Y. & Hochberg, Y. Controlling the false discovery rate: a practical and powerful approach to multiple testing. *J. R. Stat. Soc. Series B Stat. Methodol.* **57**, 289-300 (1995).

7. Tusher, V.G., Tibshirani, R. & Chu, G. Significance analysis of microarrays applied to the ionizing radiation response. *Proc. Natl. Acad. Sci. U S A.* **98**, 5116-5121 (2001).

8. Chen. J. *et al.* Practical approach for the identification and isomer elucidation of biomarkers detected in a metabonomic study for the discovery of individuals at risk for diabetes by integrating the chromatographic and mass spectrometric information. *Anal Chem.* **80**:1280-9 (2008).

**Supplementary tables:**

Table S1 The values of changing factor *q* for *k* and *ω* , which both followed exponential distributions, when inducing FDR of 0.00% at *n*=30.

| ***q* values for *k and ω*** | ***q* values for *k and ω*** |
| --- | --- |
| 0.5, 0.6 | 0.8, 0.8 |
| 0.5, 0.7 | 0.8, 0.9 |
| 0.5, 0.8 | 0.8, 1.0 |
| 0.5, 0.9 | 0.9, 0.1 |
| 0.6, 0.4 | 0.9, 0.2 |
| 0.6, 0.5 | 0.9, 0.3 |
| 0.6, 0.6 | 0.9, 0.4 |
| 0.6, 0.7 | 0.9, 0.5 |
| 0.6, 0.8 | 0.9, 0.6 |
| 0.6, 0.9 | 0.9, 0.7 |
| 0.6, 1.0 | 0.9, 0.8 |
| 0.7, 0.3 | 0.9, 0.9 |
| 0.7, 0.4 | 0.9, 1.0 |
| 0.7, 0.5 | 1.0, 0.1 |
| 0.7, 0.6 | 1.0, 0.2 |
| 0.7, 0.7 | 1.0, 0.3 |
| 0.7, 0.8 | 1.0, 0.4 |
| 0.7, 0.9 | 1.0, 0.5 |
| 0.7, 1.0 | 1.0, 0.6 |
| 0.8, 0.3 | 1.0, 0.7 |
| 0.8, 0.4 | 1.0, 0.8 |
| 0.8, 0.5 | 1.0, 0.9 |
| 0.8, 0.6 | 1.0, 1.0 |
| 0.8, 0.7 |  |

**Supplementary figures:**


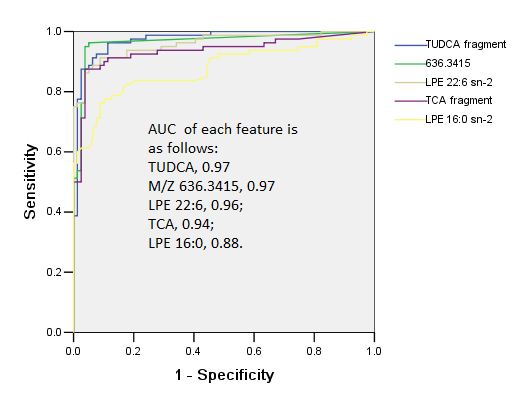


**Fig. S1 The receiver operator characteristic curves (ROCs) of the three most important features (TUDCA, the feature with m/z of 636.3415, LPE 22:6) to distinguish liver disease from control with the known biomarker TCA and LPE 16:0 included.**


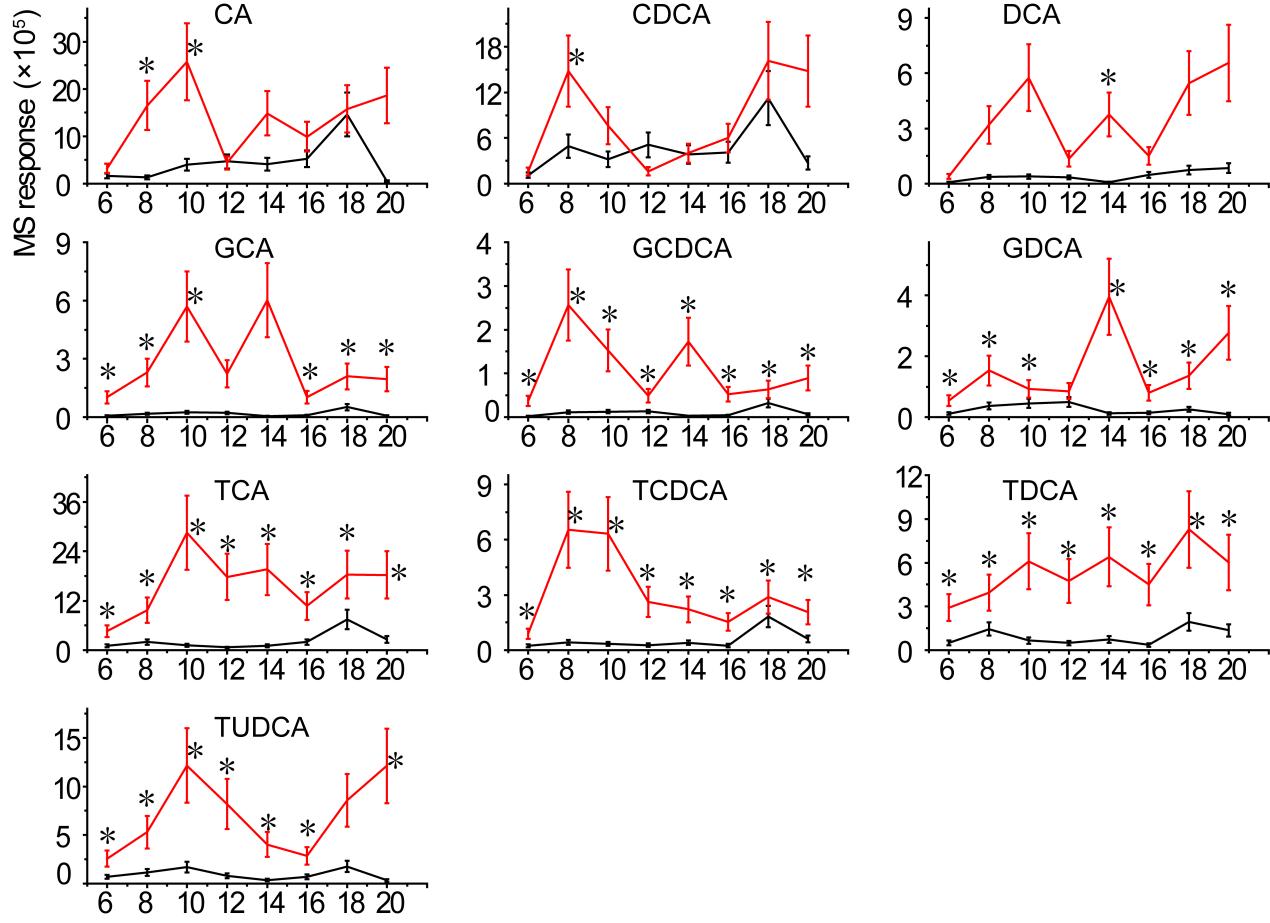


**Fig. S2 The relative contents of bile acids in model rats treated with DEN compared to those in control rats at the paired time points.** CA: cholic acid, CDCA: chenodeoxycholic acid, DCA: deoxycholic acid, GCA: glycocholic acid, GCDCA: glycochenodeoxycholic acid, GDCA: glycodeoxycholic acid, TCA: taurocholic acid, TCDCA: taurochenodesoxycholic acid, TDCA: taurodeoxycholic acid, GCDCAS: glycochenodeoxycholate sulfate, GDCAS: glycodeoxycholate sulfate, TUDCA: tauroursodeoxycholic acid, HDCA: hyodeoxycholic acid.
